# Role of Oxidative Stress in Mitochondrial Function: Relevance for Liver Function

**DOI:** 10.3390/antiox12091784

**Published:** 2023-09-20

**Authors:** Jie Xu, Zhihui Feng

**Affiliations:** 1 Center for Mitochondrial Biology and Medicine, The Key Laboratory of Biomedical Information Engineering of Ministry of Education, School of Life Science and Technology, Xi’an Jiaotong University, Xi’an 710049, China; 2 Frontier Institute of Science and Technology, Xi’an Jiaotong University, Xi’an 710049, China; 3 School of Health and Life Sciences, University of Health and Rehabilitation Sciences, Qingdao 266114, China

The traditional recognition of mitochondria as powerhouses that generate ATP and reactive oxygen species (ROS) via oxidative phosphorylation and the tricarboxylic acid cycle has ceased. Mitochondria are active and dynamic organelles that act as the hubs of cellular signaling and metabolic activity in almost all eukaryotic cells, supporting organ functions and body health. An imbalance in either part of the mitochondrial activity leads to mitochondrial dysfunction and, potentially, to oxidative stress, which directly contributes to multiple diseases. As the major metabolic organ, the liver plays a vital role in modulating whole-body metabolic health with help from its richest organelles, the mitochondria, which play central roles in hepatic glucose and lipid and protein metabolism. The vicious circle between mitochondrial dysfunction and oxidative stress is a key contributor to the progression of almost all hepatic diseases. Therefore, exploring nutritional strategies for maintaining mitochondrial homeostasis and metabolic health is as important as deciphering the mechanisms underlying hepatic diseases. Consequently, we curated this Special Issue, and identified five relevant articles, including four review articles and one research paper, presenting recent advances in the field of mitochondria and hepatic health regulation.

In Mexican traditional medicine, *Eryngium carlinae* has been used to treat lipid disorders and diabetes. Alfredo Saavedra-Molina et al. explored the effects of an ethyl acetate extract of *Eryngium carlinae* inflorescences on diabetic rat livers [1]. Their study demonstrated that the phenolic compounds of the ethyl acetate extract of *E. carlinae* inflorescences had dose-dependent antioxidant activity. The extract decreased weight loss, serum and liver TG contents, ATL, ALP, and AST levels, and inhibited reactive oxygen species (ROS) production, showing a hepatoprotective effect by restoring mitochondrial complex activity and antioxidant enzyme activity.

In a review article, Carlos M. Palmeira et al. summarized the effects of oxidative stress and polyphenols on liver disease [2]. The authors first described the effects of oxidative stress on liver disease in detail. Endogenous and exogenous sources of stress stimulate the generation of reactive oxygen species, and the cellular antioxidant system converts them to hydrogen peroxide, which participates in regulating numerous cellular functions through the KEAP1/Nrf2, IκB/NF-κB, PTEN/PI3K/AKT, and AMPK pathways. The liver is essential for multiple functions, and oxidative stress contributes to liver pathologies in which cellular redox signaling plays an important role. Polyphenols possess antioxidant and anti-inflammatory properties, and have great therapeutic potential for liver diseases. Therefore, Carlos M. Palmeira et al. also reviewed the effects of quercetin, resveratrol, and curcumin on regulating oxidative stress through preclinical and experimental studies in models of nonalcoholic fatty liver disease (NAFLD), hepatocellular carcinoma (HCC), and ischemia-reperfusion injury (LIRI).

Similarly to the previous review, Bonglee Kim et al. [3] systematically summarized the effects of traditional therapeutic herbs on oxidative stress-related liver diseases. Mitochondrial dysfunction is closely associated with multiple liver diseases. Unlike the previous review, in this article, the authors focused on alcoholic liver disease and nonalcoholic fatty liver disease. Disruption of the mitochondrial pathway increases free fatty acid transport to the liver, oxidative stress, and lipid peroxidation, which accelerates the progression of hepatitis and nonalcoholic steatohepatitis (NASH). An increase in the ratio of NADH to NAD+ increases steatosis, and cytochrome P-450 2E1 (CYP2E1) activities increase hydroxyl radicals, which are linked to the development of alcoholic liver disease (ALD). Herbal compounds reduced the mitochondria-mediated oxidative stress and ROS in liver disease. In this review, the roles of *Alisma orientalis*, *Cyclocarya paliurus*, *Lonicera caerulea* L., and other herbs are discussed together.

Cardiac hepatopathy is a special liver disease caused by cardiac dysfunction. Cardiogenic shock and hepatic circulation disorder are considered the causes of cardiogenic liver injury. However, Alexander E. Berezin et al. [4] reviewed the current research on the regulatory role of hepatokines (including fetuin-a, alpha1-microglobulin, fibroblast growth factor-21 (FGF21), and selenoprotein P) in interorgan interactions, and proposed that hepatokines serve as adaptive regulators of metabolic homeostasis in heart failure patients by counteracting oxidative stress and promoting mitochondrial dysfunction, which may play an important role in the progression of cardiac hepatopathy. The authors also proposed that FGF21 and selenoprotein P may be potential therapeutic targets for cardiogenic liver injuries.

Ketogenic diets have increased in popularity due to their health benefits. The potential benefits of this diet may be due to the alleviation of oxidative stress and the restoration of mitochondrial function. Giuseppe Cerullo et al. [5] generalized the main pathway involved in the effects of a ketogenic diet on mitochondrial homeostasis. As mentioned above, an imbalance between the antioxidant system and ROS generation leads to liver injury and dysfunction. This article also describes how chronic oxidative stress stimulates mitochondrial dysfunction and the progression of NAFLD, and how a ketogenic diet improves liver functions by promoting mitochondrial functions.

In conclusion, the articles in this Special Issue emphasize the important role of oxidative stress in hepatic mitochondrial dysfunction, and highlight treatment strategies and compounds that preserve mitochondrial function, which could lead to new avenues and approaches for improved liver health.
